# BwimNet: A Novel Method for Identifying Moving Vehicles Utilizing a Modified Encoder-Decoder Architecture

**DOI:** 10.3390/s20247170

**Published:** 2020-12-14

**Authors:** Yuhan Wu, Lu Deng, Wei He

**Affiliations:** 1 College of Civil Engineering, Hunan University, Changsha 410082, China; yuhan_wu@hnu.edu.cn (Y.W.); denglu@hnu.edu.cn (L.D.); 2 Key Laboratory for Damage Diagnosis of Engineering Structures of Hunan Province, Hunan University, Changsha 410082, China

**Keywords:** structural health monitoring (SHM), bridge weigh-in-motion (BWIM), inverse problem, convolutional neural network

## Abstract

Traffic loading monitoring plays an important role in bridge structural health monitoring, which is helpful in overloading detection, transportation management, and safety evaluation of transportation infrastructures. Bridge weigh-in-motion (BWIM) is a method that treats traffic loading monitoring as an inverse problem, which identifies the traffic loads of the target bridge by analyzing its dynamic strain responses. To achieve accurate prediction of vehicle loads, the configuration of axles and vehicle velocity must be obtained in advance, which is conventionally acquired via additional axle-detecting sensors. However, problems arise from additional sensors such as fragile stability or expensive maintenance costs, which might plague the implementation of BWIM systems in practice. Although data-driven methods such as neural networks can estimate traffic loadings using only strain sensors, the weight data of vehicles crossing the bridge is difficult to obtain. In order to overcome these limitations, a modified encoder-decoder architecture grafted with signal-reconstruction layer is proposed in this paper to identify the properties of moving vehicles (i.e., velocity, wheelbase, and axle weight) using merely the bridge dynamic response. Encoder-decoder is an unsupervised method extracting higher features from original data. The numerical bridge model based on vehicle-bridge coupling vibration theory is established to illustrate the applicability of this new encoder-decoder method. The identification results demonstrate that the proposed approach can predict traffic loadings without using additional sensors and without requiring vehicle weight labels. Parametric studies also show that this new approach achieves better stability and reliability in identifying the properties of moving vehicles, even under the circumstances of large data pollution.

## 1. Introduction

Moving vehicles are one of the main sources of live loads on bridges. Over the years, overloading has posed a significant threat to the service security and maintenance of transportation infrastructures [1,2,3]. Gathering traffic information is thus essential to the overloading and bridge health monitoring, providing a decision-making basis for evaluating the in-serve performance of bridge structures and helping regulate transportation [3,4]. Among the traffic information, the axle weights (AWs) of moving vehicles are the most difficult to obtain. A straightforward solution for obtaining the axle loads of vehicles is to install an axle load meter such as a bending beam load sensor on the road surface. However, being exposed to the impact of vehicle loads, the axle load meter is inevitably subjected to damage and require frequent maintenance. Moreover, noise and vibration also severely interfere with the performance of the axle load meter. Another approach widely adopted nowadays is the bridge weigh-in-motion (BWIM) technique weighing the moving vehicles treating the bridge as a weighing scale, which is the inverse problem of estimating the traffic loadings, given the bridge dynamic responses [5,6,7]. The bridge dynamic strain responses at one specific measurement section captured by the strain sensors installed at the underside of the bridge deck or girder are utilized by the BWIM algorithm. Besides, to get an accurate estimation of vehicles’ axle weights, additional information of vehicles, i.e., the speed and axle spacing, is also required. Thus, both strain sensors for measuring strain responses and axle-detecting devices for identifying vehicle speeds and axle spacings are required for conventional BWIM systems. Pavement sensors such as pneumatic tubes or tape switches fixed atop the bridge deck were firstly used to detect axles. While these methods have low durability due to bearing traffic loads directly [8,9,10], they are nowadays generally replaced by the more advanced free-of-axle-detector (FAD) technique, in which sensors were attached to the bridge bottom in modern BWIM systems. However, most FAD methods still have a drawback of limited application scope [8,11,12]. In addition to pavement and FAD sensors, due to the development of computer vision technology in the transportation field, vehicle attributes such as axle spacing and velocity can be easily acquired utilizing surveillance cameras [13,14,15]. Ojio et al. [16] proposed a contactless BWIM system, which gives the vehicle speed, axle spacing, and bridge deflection via video analysis using the data collected from two groups of cameras in place of strain sensors and axle detectors needed by the conventional systems. Unfortunately, additional axle detecting devices are still required for these methods and the underlying weight information concealed in the bridge dynamic responses is ignored. Once one sensor of the whole strain system fails, the weights of moving vehicles may hardly be identified. The issues existing in connection and strict synchronization among multiple sensors still imply a source of instability for BWIM systems [17].

To avoid the installation of extra axle-detecting devices, a lot of efforts have been devoted to obtaining the speed and axle spacing using only strain sensors. He et al. [18] proposed a novel virtual simply supported beam (VSSB) method exploring the axle information in the bending strain responses measured at six different sections of the bridge girder instead of one single measurement section. Then, Deng et al. [19] developed a modified method named the equivalent shear force (ESF) method, in which the number of sections needed to be measured was reduced from six to four. The drawback of the methods in He et al. [18] and Deng et al. [19] is the costly installation and the maintenance expenses associated with the number of pre-selected measuring sections.

The possibility of detecting axle information using only one specific measurement cross section (such as midspan) was also investigated. Kalhori et al. [20] applied the peak-to-peak approach to identify the vehicle axles from the bending strain time history and reported the latent defect of the approach (the uncontrolled axle measurement missing). Yu et al. [21] used the wavelet transform for the global bridge bending strain signal to detect the vehicle axles, but the accuracy of the transformation is affected by noises and dynamic effects. Takaya et al. [22] developed a mono-sensor method which utilizes a neural network to identify the transverse position, speed, and axle count of vehicles based on the bridge strain response. Although this method cannot identify the traffic loads, it makes full use of the convenience in optically measuring the speed or axle information of vehicles to create the associated dataset, providing inspiration for a stable, compact BWIM system with mono-sensor or single measuring section.

In addition to the conventional method, the axle weights (AWs) and gross vehicle weight (GVW) of vehicles can be obtained by using neural networks which act as a strong function approximator [23,24] to extract vehicle information from signals of WIM sensors or bridge responses. González et al. [25] studied the performance of a multi-layer feed-forward artificial neural network (ANN) algorithm applied to a WIM system and reported a good noise-resistant ability of ANN in WIM. The application of ANN to BWIM was early attempted by Kim et al. [26]. AWs and GVW of moving vehicles can be estimated based on the velocity and wheelbase of the vehicles, and the related bridge responses. In their study, the training data was acquired from other adjacent independent WIM systems. Takaya et al. [27] applied a deep learning method to the BWIM field in 2020, identifying properties of vehicles from bridge responses. The dataset consisted of 40,613 passing vehicles (5923 of them have known AWs obtained from a weighing station 3.6 km away). In the previous neural network studies, the sources of vehicle weight data for training are usually adjacent weighing stations or WIM systems. However, it could be challenging to obtain sufficient vehicle weight data for applying the networks at regular sites where there is no such weighing station or systems.

In this paper, a vehicle sensing system (BwimNet) utilizing a modified encoder-decoder neural network architecture is proposed, which is carefully designed so that the deficiencies inherently in the approaches mentioned above are improved. The BwimNet method detects the vehicles crossing the target bridge and estimates their properties (including velocity, axle spacings, and axle weights) using only the strain data measured at midspan without relying on extra sensors. Besides, the modified encoder-decoder architecture rebuilds the bridge dynamic responses and finds the optimal value of traffic loads heuristically without requiring supervised learning. Namely, the difficult-to-obtain vehicle weight data is not necessary for the training process of BwimNet. Numerical simulations based on vehicle-bridge coupling vibration theory and parametric studies have been conducted to demonstrate the effectiveness and applicability of the BwimNet method. The results show that the proposed method can successfully identify the properties of vehicles including speed, axle count, axle spacing, and axle weight with good accuracy and robustness.

The main content of the paper is organized as follows. The proposed BwimNet method is introduced in the next section. The numerical method and the vehicle-bridge coupling vibration model are explained in Section 3. The results of simulation and case studies are presented in Section 4. Finally, the conclusions are summarized in Section 5.

## 2. Proposed BwimNet Method

Conventional BWIM systems identify the wheelbase, speed, and axle count via axle detectors, and then estimate the traffic loadings through consulting the obtained wheelbase and speed of moving vehicles. In comparison, the new traffic loading monitoring technique introduced in this section predicts the vehicles’ axle spacing, speed, and weight through mining only the bridge dynamic responses data.

Convolutional neural network (CNN) is an effective data mining algorithm, which is a type of neural network with convolutional layers and pooling layers [23,28]. Because of convolutional layers, CNN has fewer network parameters and a stronger feature learning ability than the backpropagation (BP) neural network. In recent years, CNN has achieved a prominent performance in image recognition, object detection, and some other fields such as structure health monitoring [29,30,31,32,33].

In BwimNet, two convolutional neural networks (denoted as CNN-1 and CNN-2) were utilized to predict vehicles’ axle count and estimate their properties (vehicle weights, velocity, and axle spacings), respectively. Structures of CNN-1 and CNN-2 are represented in Appendix A. Since the size of CNN-2′s output layers (predicting axle spacings and AWs) are related to the value of axle count, CNN-1 should be applied to estimate the axle count of vehicles before identifying their other properties by using CNN-2. In this study, the sum of strains, a one-dimensional sequence, recorded by strain sensors mounted on the soffit of each girder were taken as the input of CNN-1 and CNN-2. For the sake of training convergence, the input signals should be normalized in advance by the min-max normalization method, namely, the signals are scaled into sequences ranging from 0 to 1 before being fed into those convolutional neural networks. The training data of axle spacings and velocity can be obtained from surveillance cameras or temporary devices for detecting speed and axle spacings, and the proposed method can be trained without the data of vehicle weight due to a specially designed encoder-decoder architecture. A more detailed description of the input signal and training data can be found in Table 1.

### 2.1. Axle Count Predicting

Since the axle count varies from vehicle to vehicle, obtaining the axle count of vehicles in advance would be of assistance to the identification of other properties. In BwimNet, a one-dimensional convolutional neural network denoted as CNN-1 is used to classify the vehicles into several categories according to their axle counts. Bridge response signals were the input of CNN-1. The most possible axle count is output. In the classification, the one-hot encoding scheme was adopted to encode the possible axle counts of the vehicle, a group of limited integers, into a batch of binary vectors indicating the information of categorical data. The widely-used cross entropy function was then adopted as the loss function of CNN-1, which can be expressed as follows:

(1) $${ℒ}_{1}=-\sum_{i=1}^{N}y_{i}\log({\hat{y}}_{i})$$

where N = number of categories (each category contains vehicles with same axle count); $y_{i}$ = actual category (encoded to one-hot vector); ${\hat{y}}_{i}$ = the network score for the *i*th category. More detailed information on CNN-1 can be found in Appendix A. From Equation (1), it can be seen that ${ℒ}_{1}$ goes down when the right category gets a higher score and the other categories get a lower score. Therefore, the axle count of the vehicle can be obtained by finding out the minimum of the loss function.

### 2.2. Velocity and Wheelbase Predicting

**Figure 1:**
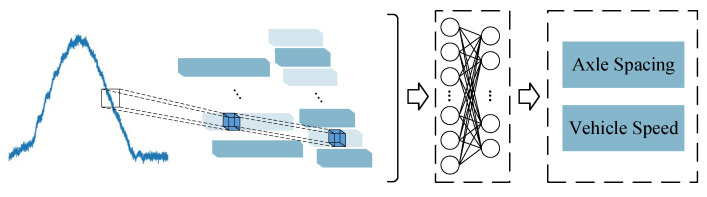
Framework of the velocity and wheelbase identification.

As shown in Figure 1, another convolutional neural network denoted as CNN-2 is utilized to identify the speed and axle spacings of moving vehicles based on the information of bridge responses. Three CNN-2 models with different dimensions of output layers were designed for the 2-axle, 3-axle, and 5-axle truck models adopted in this paper, respectively. The detail of CNN-2 is shown in Appendix A. Based on the actual axle count of vehicles, the vehicle datasets (including velocity and axle spacing) can be divided into several categories according to their axle count. Then, the three CNN-2 models will be trained using the dataset with the corresponding axle count. The loss functions of speed and axle spacing prediction are shown in the following formulas:

(2) $${ℒ}_{2}={\left\|\;v_{}^{m}-v_{}^{p}\right\|}_{}^{2}$$

(3) $${ℒ}_{3}=\frac{\sum_{i=1}^{N-1}{\left\|\;d_{i}^{m}-d_{i}^{p}\right\|}_{}^{2}}{N-1},$$

where $v_{}^{m}$ = actual speed of vehicles; $v_{}^{p}$ = predicted speed of vehicles; $d_{i}^{m}$ = actual axle spacing between the first axle and the *i*th axle; $d_{i}^{p}$ = predicted axle spacing between the first axle and the *i*th axle; *N* = count of vehicle axles.

### 2.3. Vehicle Weight Predicting

**Figure 2:**
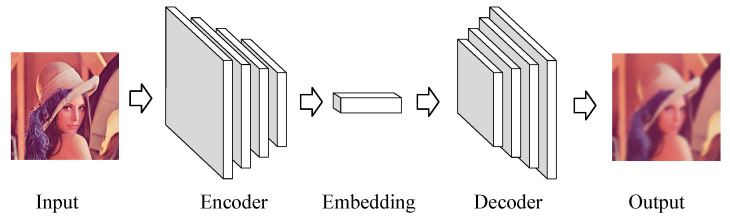
Structure of a typical encoder-decoder network.

The encoder-decoder network [34,35] is a widely-adopted unsupervised learning method, having been proved effective in computer vision, natural language processing, etc. As shown in Figure 2, an auto-encoder architecture is composed of an encoder and a decoder, learning latent attributes from the input data. The encoder is a neural network that transforms the input (i.e., an image or sequence) into a compressed feature vector named embedding, and the decoder then gradually recovers the image or sequence from the extracted embedding. One advantage of the encoder-decoder method is its outstanding performance in representation learning as an unsupervised learning algorithm by minimizing the difference between the original input and the reconstructed output.

Although utilizing an unsupervised encoder-decoder method can learn latent features such as traffic loading information from the bridge responses, the extracted embedding containing underlying loading information is just a sparse vector and can hardly be understood. To make the embeddings explainable, modification is required for the encoder-decoder method. Conventional BWIM systems adopt a linear assumption of the bridge responses, where the strain at the time t is equivalent to the production of the pre-calibrated bridge influence line IL and the traffic loadings F:

(4) $$\varepsilon (t)=\int_{0}^{l}IL(x)\cdot F(x-v\cdot t)dx,$$

where l = span of the instrumented bridge. It should be noted that by considering **IL**(*x*) is a compactly supported function on [0, *l*] and let f(t) = IL(v⋅t) and g(t)=v⋅F(−v⋅t), Equation (4) can be rewritten as a convolutional process:

(5) $$\varepsilon (t)=\int_{-\infty }^{+\infty }f(\tau )\cdot g\left(t-\tau \right)d\tau .$$

We designed a signal-reconstruction layer based on the convolutional process shown in Equation (5) and grafted it onto an encoder-decoder method to take the place of an ordinary decoder. Unlike an unexplainable “black box” decoder, the physical significance of the signal-reconstruction layer is clear, which rebuilds the bridge response signal by consulting the information about vehicle and bridge. The encoder can be treated as a strong feature extractor that directly identifies the traffic loadings (the output of the encoder is an approximate solution to the inverse problem that *F*(*x*) in Equation (4) is needed to be determined) merely based on the bridge dynamic responses.

**Figure 3:**
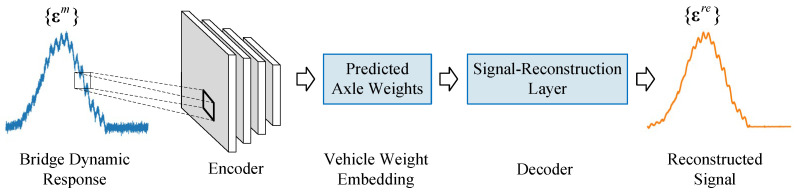
Architecture of BwimNet (modified encoder-decoder network).

Figure 3 demonstrates the architecture of the modified encoder-decoder method in the BwimNet, which helps optimize parameters of the encoder without knowing the actual vehicle weight.

In the whole training process, datasets used in velocity and wheelbase predicting are required in the decoder. The reconstructed signal $\left\{\varepsilon^{r}\right\}$ is calculated by the predicted axle weights $\left\{F^{p}\right\}$, calibrated influence line, and associated vehicle speed and axle spacing. The reconstructed signal at time step *k* $\varepsilon_{k}^{r}$ can be obtained as follows:

(6) $$\varepsilon_{k}^{r}=\sum_{i=1}^{N}F_{i}^{p}I(v\cdot t_{k}-d_{i}),$$

where v = vehicle speed; $d_{i}$ = distance between the first axle and the *i*th axle; I = the instrumented bridge’s strain influence line which can be obtained by the calibration method proposed by O’Brien et al. [6]; $F_{i}^{p}$ = axle weight of the *i*th axle predicted by the neural network. The loss function of the encoder can be defined as the Euclidean distance between the measured signal $\left\{\varepsilon^{m}\right\}$ and the reconstructed signal $\left\{\varepsilon^{r}\right\}$, which can be expressed as follows:

(7) $${ℒ}_{4}={\left\|\left\{\varepsilon^{m}\}-\{\varepsilon^{r}\right\}\right\|}_{2}^{2}.$$

With training epochs increasing, the reconstructed signal $\left\{\varepsilon^{r}\right\}$ will gradually approach the measured signal $\left\{\varepsilon^{m}\right\}$, and thus the predicted AW will gradually get close to the actual AW. During the whole training process, the dataset of AW is not required and, instead, datasets utilized in velocity and wheelbase predicting are needed, which can be obtained easily through surveillance cameras or just by installing some temporary devices. Since the axle weight identification shares the same datasets with the estimation of vehicle velocity and axle spacing, the multiple tasks learning method is utilized to predict the AW, wheelbase, and speed in one neural network model (CNN-2).

**Figure 4:**
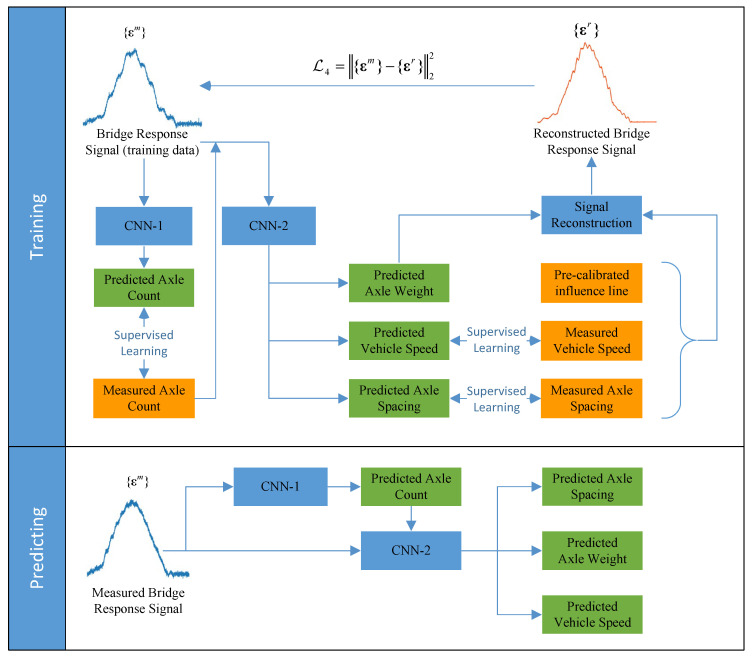
Framework of the proposed method. CNN-1, the neural network predicting axle count; CNN-2, the neural network predicting axle weight, axle spacing, and speed.

The whole process of the BwimNet method is described by the flow chart shown in Figure 4. The detailed structures of the networks are illustrated in Appendix A, and detailed implementation procedure of BwimNet is shown in Appendix C. In this study, the identification error *e* of axle spacings, axle weights, and speed of different vehicles was defined as follow:

(8) $$e=\left|\frac{A_{\mathrm{iden}}-A_{\mathrm{true}}}{A_{\mathrm{true}}}\right|\times 100\%$$

where $A_{\mathrm{iden}}$ and $A_{\mathrm{true}}$ are then identified and the true vehicle attribute (axle spacing, axle weight, or speed), respectively.

After the whole training processes of CNN-1 and CNN-2 are finished, the axle count of the vehicle to be measured can be estimated based on their bridge responses at first. Then, the corresponding CNN-2 with the correct dimension of output layers is chosen based on its axle count. Finally, the speed, axle spacings, and axle weights of the target vehicle can be predicted from the bridge strain response using the selected CNN-2. In the process of vehicle attributes predicting, the input only contains strain responses of the bridge, which eliminates the need for extra sensors, cameras, or temporary devices for detecting speed and axle spacings.

## 3. Numerical Simulation

When the vehicle passes a bridge, the impact of vehicle speed and axle loads will lead to the vibration of the bridge structure. Meanwhile, the vibration of the bridge will feedback on the dynamic behavior of moving vehicles in turn. In addition, the existence of bridge road surface roughness and the impact of the bridge approach will aggravate the vibration of vehicle and bridge. The vehicle-bridge coupling vibration theory is a method investigating the interaction between the dynamic behavior of bridge and vehicle [36].

In this paper, a computer program based on Ansys APDL and MATLAB platform, which had been validated through field tests [37,38,39], was adopted to numerically simulate the bridge dynamic responses under traffic loads instead of a simple influence line loading approach. The simulated bridge dynamic responses were then used for identifying vehicle properties by the proposed method.

### 3.1. Vehicle-Bridge Coupling Vibration Model

#### 3.1.1. Road Surface Condition

In recent years, a lot of studies about vehicle-bridge coupling vibration have been conducted. Deng et al. and Yang et al. [40,41] made detailed reviews on the development and application of vehicle-bridge coupling theory. One of the main excitation sources for vehicle-bridge coupling vibration is the road surface roughness, which can be described as follows [42,43]:

(9) $$\left\{ \begin{array}{l} r(x)=\sum_{k=1}^{N}\sqrt{2\varphi \left(n_{k}\right)\Delta n}\cos\left(2\pi n_{k}x+\theta_{k}\right)\\ \varphi (n)=\varphi \left(n_{0}\right){\left(\frac{n}{n_{0}}\right)}^{-2}\left(n_{1}<n<n_{2}\right) \end{array}\right.$$

where $\theta_{k}$ = random phase angle obeying uniform distribution between 0 and 2π; φ() = power spectral density (PSD) function for the road surface elevation (m^3^/cycle/m); $\varphi (n_{0})$= road surface roughness coefficient (m^3^/cycle); $n_{k}$ = wave number (cycle/m); n = spatial frequency (cycle/m); $n_{0}$ = discontinuity frequency of 0.5π (cycle/m); $n_{1}$ and $n_{2}$ = lower and upper cut-off frequencies. Based on the classification of road surface condition (RSC) proposed by the International Organization for Standardization [44], four road surface roughness coefficient 0, $5\times 10^{-6}$, $20\times 10^{-6}$, and $80\times 10^{-6}$ m^3^/cycle are adopted, which correspong to four different RSCs; smooth, very good, good, and average, respectively.

#### 3.1.2. Road Surface Condition

The dynamic equation of vehicle and bridge can be described as the following equation set:

(10) $$\left\{ \begin{array}{l} M_{b}{\ddot{d}}_{b}+C_{b}{\dot{d}}_{b}+K_{b}d_{b}=F_{b}\\ M_{v}{\ddot{d}}_{v}+C_{v}{\dot{d}}_{v}+K_{v}d_{v}=F_{v}+F_{G} \end{array}\right.,$$

where $M_{b}$, $C_{b}$, $K_{b}$, $M_{v}$ $C_{v}$, and $K_{v}$ = mass, damping, and stiffness matrices of bridge and vehicle, respectively; ${\ddot{d}}_{b}$, $d_{b}$, ${\ddot{d}}_{v}$, ${\dot{d}}_{v}$, and $d_{v}$ = acceleration, speed, and displacement vector of bridge and vehicle, respectively; $F_{b}$, $F_{v}$ = interaction forces vector between bridge and vehicle; $F_{G}$ = vector of vehicle’s gravity force.

Based on Equation (10) and the relationship of interaction force and displacement between bridge and vehicle, the equation of vehicle-bridge coupling system can be founded as follows [39]:

(11) $$\begin{array}{l} \left[\begin{array}{cc} M_{b} & \\ & M_{v} \end{array}\right]\left\{\begin{array}{l} {\ddot{d}}_{b}\\ {\ddot{d}}_{v} \end{array}\right\}+\left[\begin{array}{cc} C_{b}+C_{b-b} & C_{b-v}\\ C_{v-b} & C_{v} \end{array}\right]+\left\{\begin{array}{c} {\dot{d}}_{b}\\ {\dot{d}}_{v} \end{array}\right\},\\ +\left[\begin{array}{cc} K_{b}+K_{b-b} & K_{b-v}\\ K_{v-b} & K_{v} \end{array}\right]\left\{\begin{array}{c} d_{b}\\ d_{v} \end{array}\right\}=\left\{\begin{array}{c} F_{b-v}\\ F_{G}+F_{v-r} \end{array}\right\} \end{array}$$

where $C_{b-b}$, $C_{b-v}$, $C_{v-b}$, $K_{b-b}$, $K_{b-v}$, $K_{v-b}$, $F_{b-v}$, and $F_{v-r}$ are coefficient matrix about the interaction forces. Modal superposition and Newmark-*β* algorithm were adopted to solve the above equation. Field measurements were carried out to validate the vehicle-bridge coupling theory [37,38].

Once the dynamic displacement responses of the bridge are obtained, the bending strain of the bridge {ε} can be calculated through the following formulation:

(12) $$\{\varepsilon \}=\left[B\right]\left\{d_{b}\right\}$$

where [B] = bridge strain-displacement relationship matrix; $\left\{d_{b}\right\}$ = bridge displacement vector.

### 3.2. Simulation Setup

#### 3.2.1. Vehicle Model

**Figure 5:**
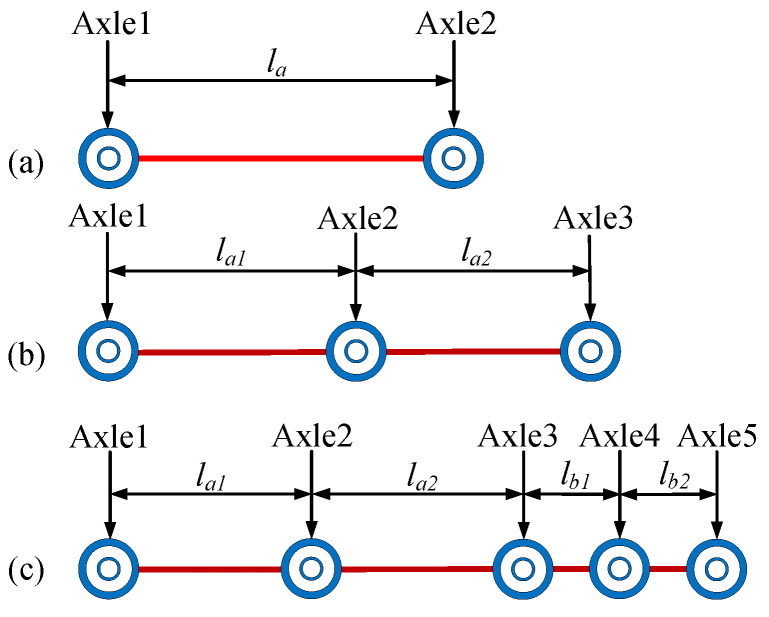
Adopted truck model: (**a**) 2-axle truck model; (**b**) 3-axle truck model; (**c**) 5-axle truck model.

Based on the previous studies on vehicle models of the vehicle-bridge coupling vibration theory, three different vehicle models shown in Figure 5 were adopted, whose axle count were 2, 3, and 5, respectively [18,45,46,47]. Based on the study on the real WIM statistical data [48,49], the axle spacing of vehicles can be divided into two categories: spacings between axles not in the same group ranging from 210 cm to 900 cm (*l_a_*) and tandem axle spacings ranging from 102 cm to 185 cm (*l_b_*).

**Figure 6:**
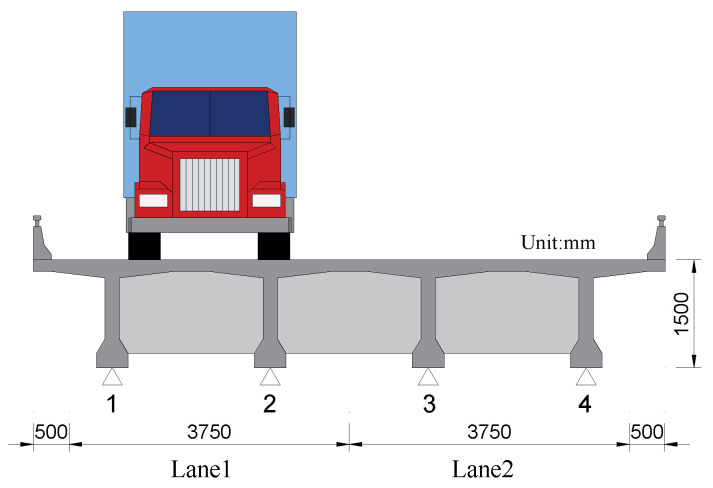
Loading position of vehicles and bridge cross-section.

In this paper, the three truck models shown in Figure 5 were adopted in numerical simulation, whose GVW ranges from 10 t to 60 t. These truck models were set to pass through the bridge model shown in Figure 6 with speeds ranging from 10 m/s to 30 m/s. Based on the vehicle-bridge coupling vibration equations mentioned above, the response signals of the bridge and corresponding datasets can be obtained.

#### 3.2.2. Bridge and Truck Models

To demonstrate the accuracy and reliability of the proposed method, a vehicle-bridge coupling model was established for numerical simulation. A simply-supported bridge model composed of 4 identical T-girders was established, which has a span of 20 m and a width of 8.5 m. Figure 6 shows the cross section of the bridge model. As shown in Figure 6, the vehicle model is loaded at the center of Lane1, and the strain signals from the bottom of the four T-girders’ midspan are collected and taken as the input of the BwimNet method.

**Figure 7:**
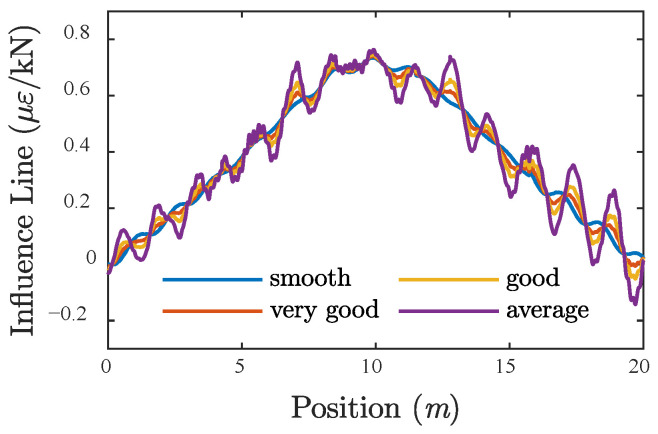
Influence line under different RSCs.

In the setting of the proposed BWIM systems, the influence lines of bridge response were obtained before training the networks. During the calibration of influence line, three trucks [46,50,51] with known attributes (listed in Table 2) are set to pass the instrumented bridge at fixed speeds several times, respectively. Then, the influence line for each run can be back-calculated based on the measured bridge responses by using the method proposed by O’Brien et al. [6]. The nine influence lines were then averaged for the following identification procedures. Figure 7 shows the four different strain influence lines of the bridge under four different road surface conditions (smooth, very good, good, and average).

#### 3.2.3. Training and Evaluation Datasets

In this study, 5000 truck samples with varied speeds and axle spacings were generated for 2-axle, 3-axle, and 5-axle trucks shown in Figure 5, respectively, and the bridge strain response induced by each truck model was acquired. It should be noted that, to make the simulation more realistic, the obtained response signals were polluted by adding 5% white gaussian noise (definition of noise level can be found in Appendix B) before being fed into the BwimNet model. Based on the collected strain data and corresponding vehicle attributes, two datasets were prepared for the two convolutional neural networks (CNN-1 and CNN-2). The *i*th sample of the dataset for CNN-1 can be described as $\left(\varepsilon_{i},N_{i}\right)$, and $\left(\varepsilon_{i},F_{i},v_{i},D_{i}\right)$ is used to describe that of CNN-2, where $\varepsilon_{i}$ is the strain data sequence, $N_{i}$ is the count of axles, $F_{i}$ is the AW of vehicle, $v_{i}$ refers to vehicle speed, and $D_{i}$ refers to axle spacing of vehicle.

For CNN-1, the dataset contained 5000 × 3 = 15,000 records of data, and the records were shuffled randomly before training. The whole dataset was then divided into a training set containing 10,000 samples and a testing set with 5000 samples. For CNN-2, to enhance the performance of AW identification, the attributes of 2-axle, 3-axle, and 5-axle truck models needed to be identified separately. Thus, three CNN-2 models with different output layers were separately trained to identify the three truck models with different axle count. Therefore, three CNN-2 datasets were prepared for the three truck models and each CNN-2 dataset contains 5000 samples, which was divided into a training set with 4000 samples and a testing set with 1000 samples.

## 4. Results

**Figure A1:**
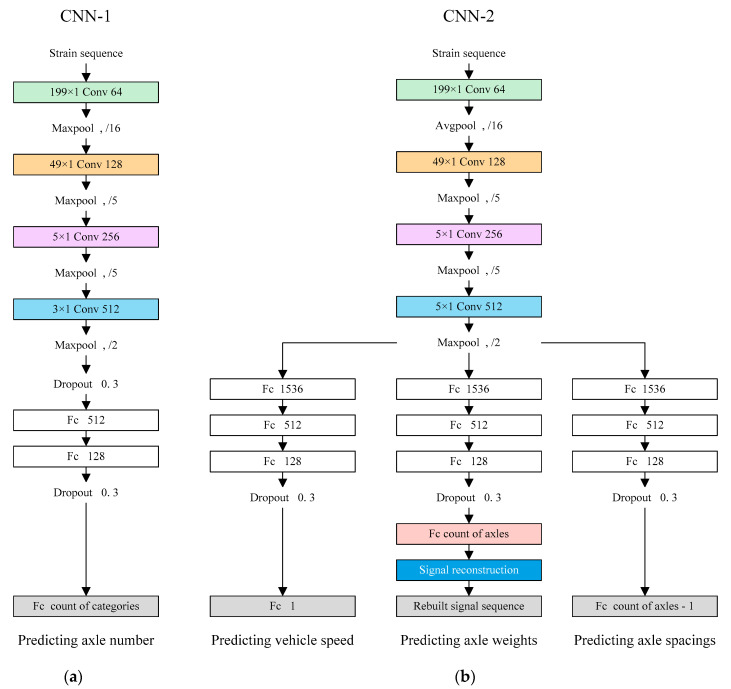
Structure of convolutional neural networks: (**a**) structure of the axle count predicting neural network; (**b**) structure of the neural network predicting speed, axle weights and axle spacings of vehicles. Conv, Convolutional layer; Fc, Fully connected layer; Coefficient of L2 regularization = 10^−5^, Learning rate = 10^−4^, Dropout coefficient = 0.3.

The neural network models (CNN-1 and CNN-2, shown in Figure A1) were written in Python 3.7 and implemented on Tensorflow 1.14, the training process, which was accelerated by invoking CUDA 10.1. The training process ended when the testing loss reaches its minimum. The performance of the BwimNet method was evaluated by comparing the prediction of testing sets with the actual value after the model training had finished. The effects of RSC, error in the training set, size of the training set, and lateral positions on the accuracy of the BwimNet method were investigated. Furthermore, a comparison between the conventional BWIM algorithm and the BwimNet method was also performed.

### 4.1. Comparison with Conventional BWIM Algorithm

The AW identification performances of the BwimNet method and conventional BWIM algorithm under good RSC for the three truck models were summarized in Table 3. It should be noted that the vehicle speed, axle spacing, calibrated influence line, and bridge response are the input of the conventional algorithm, while the input of the proposed method contains the bridge response only. As can be seen from Table 3, the BwimNet method achieves good accuracy in predicting both AW and GVW. Besides, it should be noted that the conventional BWIM algorithm shows poor performance in predicting the weights of the three rear axles of five-axle trucks. The reason may be that a lot of 5-axle trucks in the test have close-spaced axles (axle tandem) which will lead to ill-conditioned coefficient matrix in the equation for predicting axle weights [52,53]. While in the BwimNet method, the problem of ill-conditioned matrices was improved due to the contribution of dropout layer and L2 regularization, so that a higher identification accuracy can be achieved when the axles are closely spaced.

### 4.2. Effect of Road Surface Condition

**Figure 8:**
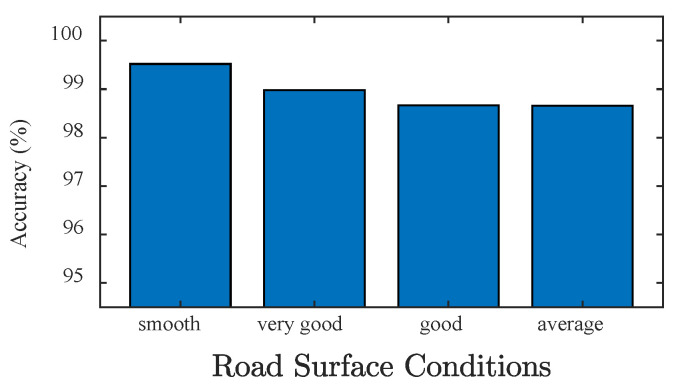
Identification performance of axle count predicting under different RSCs.

As a result of traffic loads and natural erosion, the road surface condition will gradually deteriorate during the bridge service life. The deterioration of the road surface condition also exacerbates the vehicle-bridge coupling vibration [54]. Figure 8 demonstrates the identification accuracy of the axle count under different RSCs. It can be seen that the RSC has a slight influence on the axle count identification accuracy of the BwimNet method, which means that the proposed method is robust to different RSCs.

**Figure 9:**
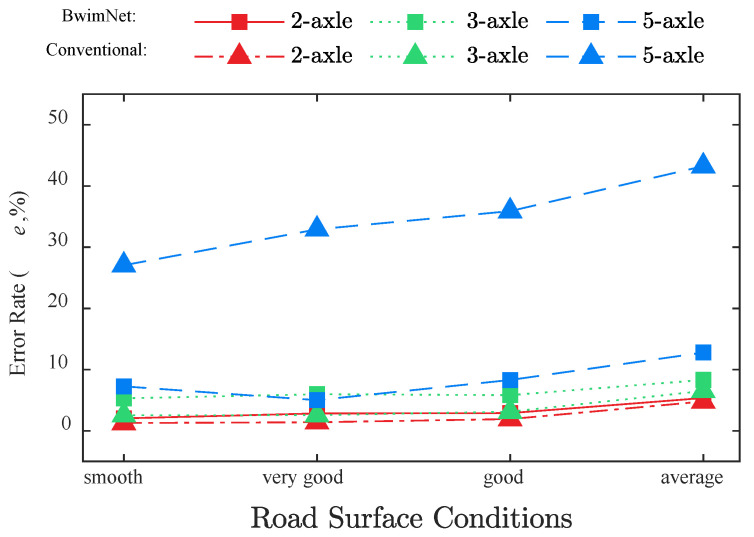
Axle weights identification accuracies of the proposed method and the conventional method under different RSCs.

The errors of AW identification for the BwimNet method and conventional BWIM algorithm under different RSCs are illustrated in Figure 9. As Figure 9 shows, there is a clear increasing trend of AW identification error for both methods when the road surface roughness worsens. Therefore, regular maintenance of road surface can be a contribution to the BWIM methods. In addition, both the BwimNet method and conventional BWIM algorithm have a good performance in identifying AW of 2-axle and 3-axle trucks. For these trucks, the identification errors of BwimNet are slightly higher than those of the conventional BWIM algorithm. While, for the 5-axle trucks, the BwimNet method has a much better performance than the conventional method. The significant errors of the conventional method occurred mainly because a lot of 5-axle trucks have close-spaced axles, which led to ill-conditioned problems when using Moses’ algorithm. The reason why the errors of the proposed BwimNet did not increase rapidly for the 5-axle trucks with close-spaced axles may be that the errors were largely reduced by the inner dropout layers and L2 regularization.

**Figure 10:**
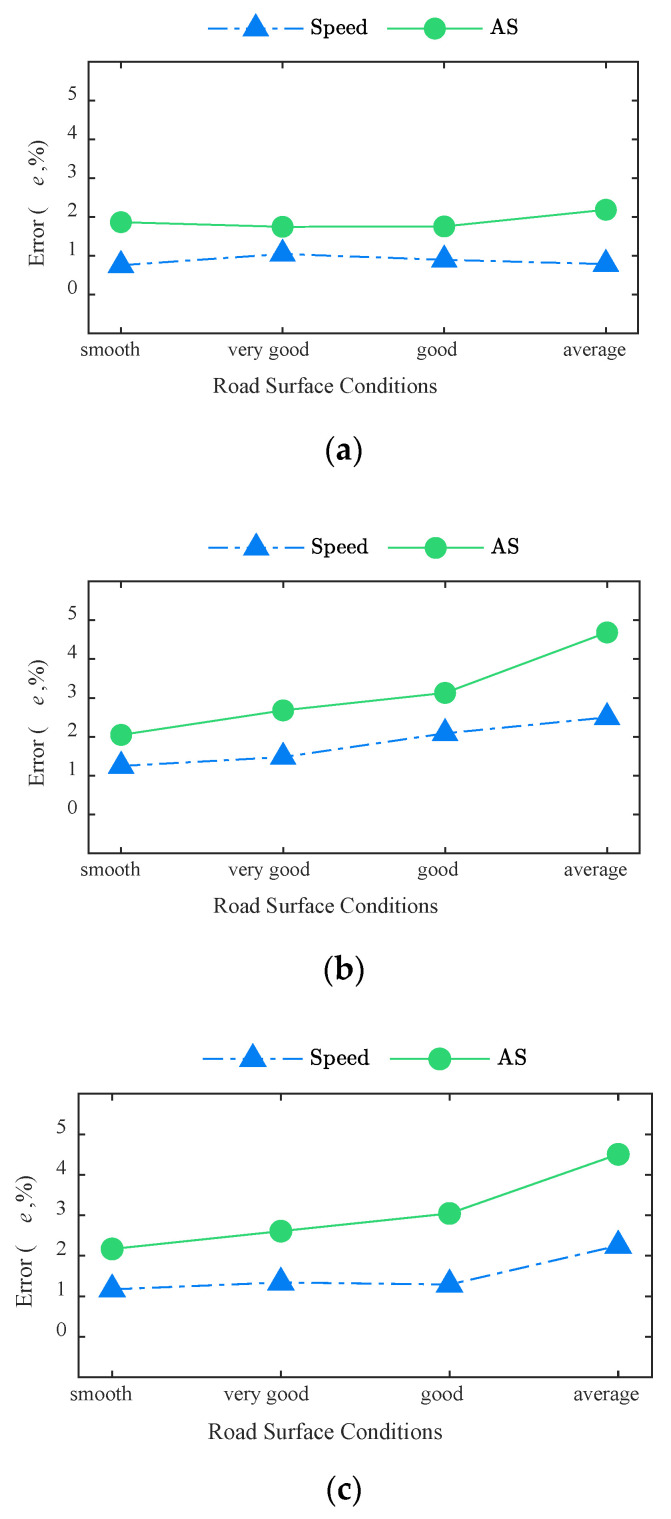
Identification performance of axle spacings and speed under different RSCs: (**a**) 2-axle trucks; (**b**) 3-axle trucks; (**c**) 5-axle trucks. AS, axle spacing.

Figure 10 shows errors of the BwimNet method in identifying speed and axle spacing under different RSCs. As can be seen from Figure 10, the BwimNet method shows a good performance in identifying axle spacing and speed of different vehicles. Same as AW identification, the errors in the identification of axle spacing and speed increase with the road surface condition, generally becoming worse. The reason that the identification errors increase when RSC becomes rougher, might be due to the fact that the intensified vehicle-bridge coupling vibration obscures some features in bridge response signals and results in a worse recognition result of the BwimNet method.

### 4.3. Effect of Error in Training Set of Speed and Wheelbase

Errors are inevitable in acquiring the training datasets of speed and axle spacings. In this paper, five ranges of error in training data, including 0%, −2%~2% (±2%), −4%~4% (±4%), −6%~6% (±6%), −8%~8% (±8%), −10%~10% (±10%), were chosen to describe the level of speed and axle spacing errors. The identification performance of the BwimNet method under different levels of error in training data was studied. The loading case of 2-axle trucks moving on good RSC was adopted to demonstrate the effects of errors in speed and axle spacing on the BwimNet method.

**Figure 11:**
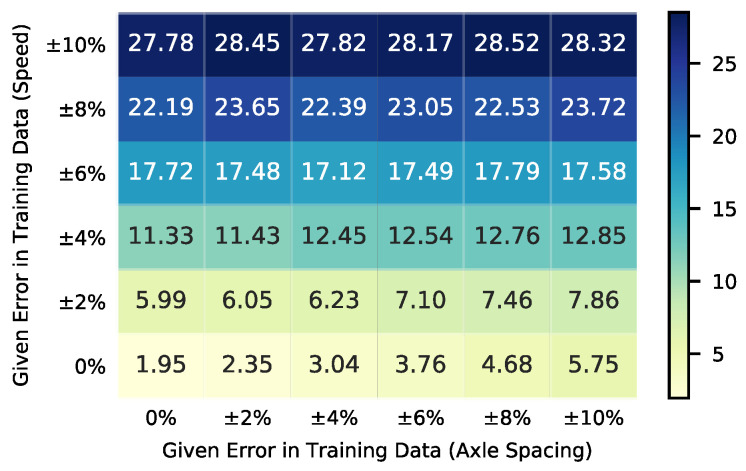
Identification error (%) of axle weights of conventional BWIM algorithm with error-included speed and axle spacing information (Two-axle Trucks).

For conventional BWIM systems, error-included vehicle speed and wheelbase data have a huge negative impact on the identification of AWs. The effects on the conventional BWIM algorithm were summarized in Figure 11. As can be seen from Figure 11, identification errors of the conventional BWIM algorithm increase significantly with the increase of speed error. Even when the speed error is as small as 4%, the weight error can reach 12%.

**Figure 12:**
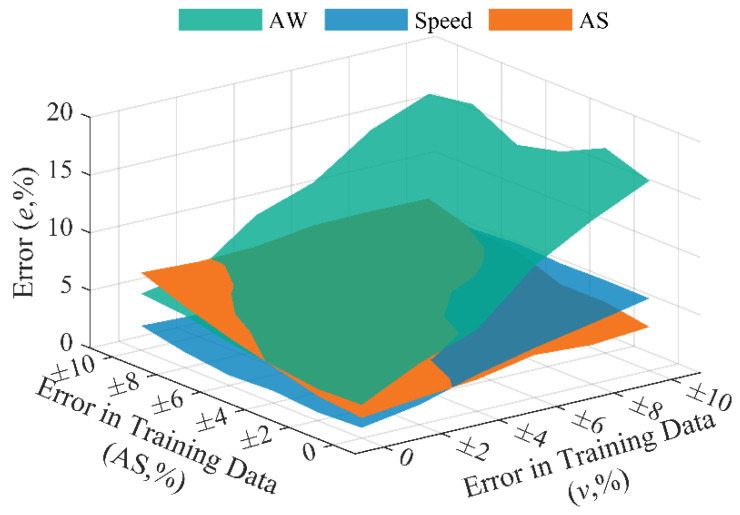
Identification error (%) of BwimNet with error-included training data of speed and axle spacing (Two-axle Trucks).

Figure 12 shows changes in identification errors of axle weight, speed, and axle spacing of the BwimNet method when errors in training data of speed and axle spacing exist. As can be seen from Figure 12, the identification error in axle weight increases significantly with the error in training data of speed increasing. While the effects of error in axle spacing are relatively smaller than that of velocity error. It should be noted that the inputs of conventional BWIM are the bridge responses, the error-included speeds, and axle spacings; as for the BwimNet method, the error-included speed and axle spacings only participate in the training process, and only the bridge responses are needed after training. BwimNet’s identification errors in speed and axle spacing slightly increase with error in training data of speed increasing. In addition, with the error in axle spacing increasing, the axle spacing identification error increases while the speed identification error tends to level off. This might be due to the fact that the speed and axle spacing identification might share some neurons in the feature map because of multiple-task learning and that the speed features in strain signals are more significant than the axle spacing features. On the other hand, for conventional BWIM systems using FAD sensors, the BWIM systems usually identify the vehicle speed in advance and then calculate the axle spacing based on the identified speed. Therefore, this phenomenon also conforms to our empirical judgment.

### 4.4. Influence of Overfitting in BwimNet and Solution

**Figure 13:**
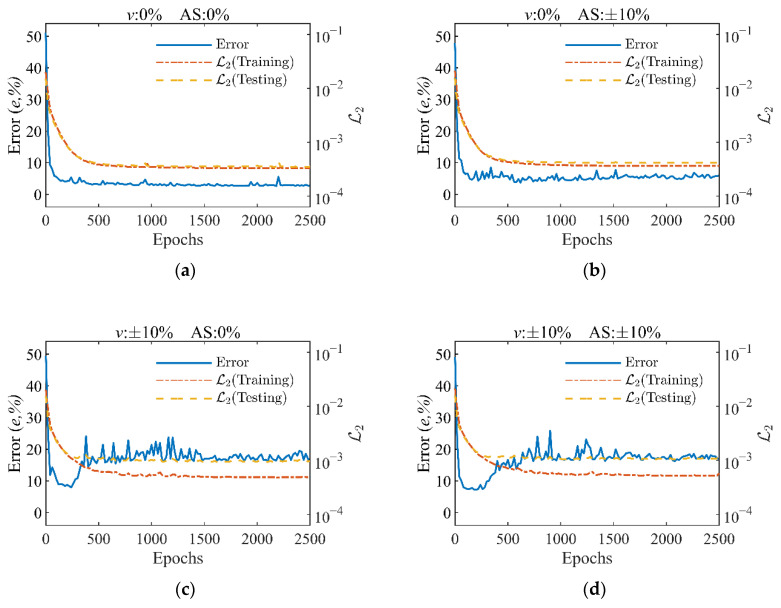
Time history curve of loss function and error during training: (**a**) no error in training data; (**b**) no error in training data of speed and given error in training data of axle spacing ranging from −10% to +10%; (**c**) given error in training data error of speed ranging from −10% to +10% and no error in training data of axle spacing; (**d**) given error in training data of speed ranging from −10% to +10% and given error in training data of axle spacing ranging from −10% to +10%. *v*, the level of error in training data of speed; AS, the level of error in training data of axle spacing.

The reason why large error exists in the traffic loads identification of BwimNet can be found in Figure 13 which shows changes of the loss function and AW identification error during the training process of the BwimNet method under four different levels of error in training data of velocity and axle spacings. The results demonstrate that when the given error in training data of speed increased to ±10%, the phenomenon of over-fitting appears where the testing loss is hard to decrease while the AW identification error decreases first and then goes up. That is most likely because when the speed error is large, the actual AW cannot minimize the difference between the reconstructed signal and the original signal. Hence, both BwimNet and conventional BWIM can hardly estimate the correct traffic loadings under that circumstance.

In order to improve the performance of BwimNet under such a tough situation, the technique of an early stop can be utilized. Since we do not have the weight of moving vehicles in the training set and testing set, it is difficult to evaluate BwimNet’s identification performance of AWs during training when significant error exists in training dataset. However, a small calibration set with extra weight information may be helpful to determine the value of some hyperparameters of the BwimNet model more wisely. For instance, BwimNet can end the training process at an early stage before overfitting appears by consulting its identification performance on the calibration set.

**Figure 14:**
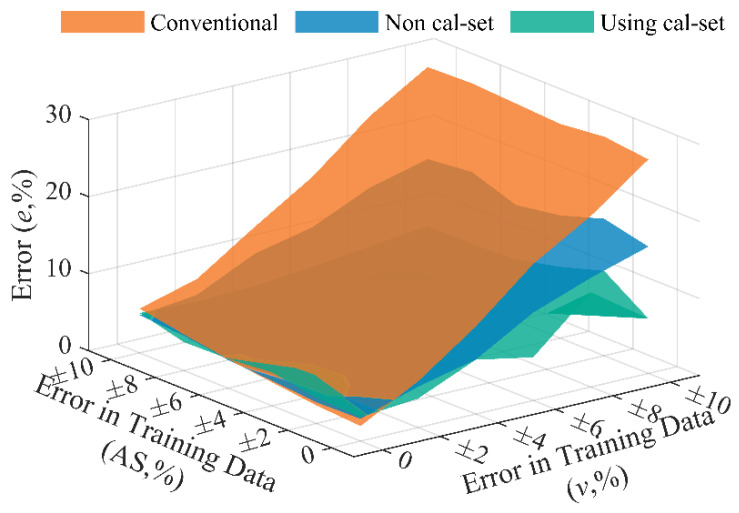
Axle weight identification error (%) of BwimNet using a calibration set. Cal-set, the abbreviation of calibration set.

Figure 14 lists the axle weight identification results of BwimNet using a calibration set with nine pieces of elements (three calibration trucks with known weight listed in Table 2 run at 10 m/s, 20 m/s, 30 m/s, respectively). As can be seen from Figure 14, when the error in training data of speed is small, the BwimNet method with a calibration set has a similar AW identification performance as without it; with the increase of error in training data of speed, the early stop technique enables the BwimNet to work properly with polluted training data, which is quite common in reality, even the calibration set consisted of very few elements (only nine pieces of element), making the BwimNet more reliable and stable in a rough working condition.

### 4.5. Effect of Size of Training Set

**Figure 15:**
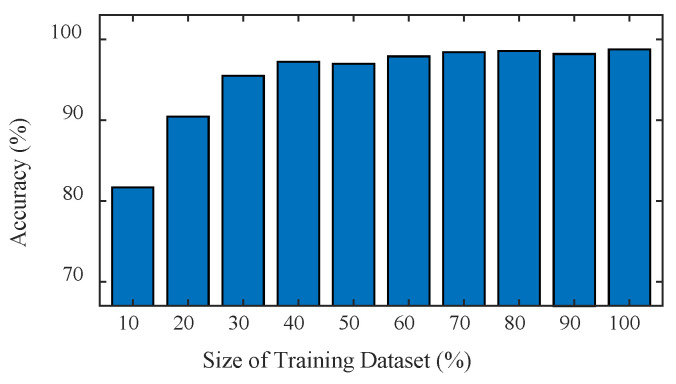
Effect of training data size on axle count classification. 100% size of training set = 10,000 samples (CNN-1).

The size of the training set also has an important influence on the performance of neural networks and a larger size of the training set will give neural networks a stronger generalization ability. Therefore, this paper studied the performance of the BwimNet method when the training set’s size was subsampled to 10~100% of the original one (CNN-1:10,000 samples; CNN-2:4000 samples) while the testing set size remains unchanged. Figure 15 demonstrations changes in axle count classification accuracy with the increase of training data size, from which it can be seen that there is a large identification error in identifying the axle count with insufficient training data and that the identification accuracy of the axle count increases significantly with the increase of training data size and reaches its saturation when the size of the training set is 40% (4000 samples) of the original one.

**Figure 16:**
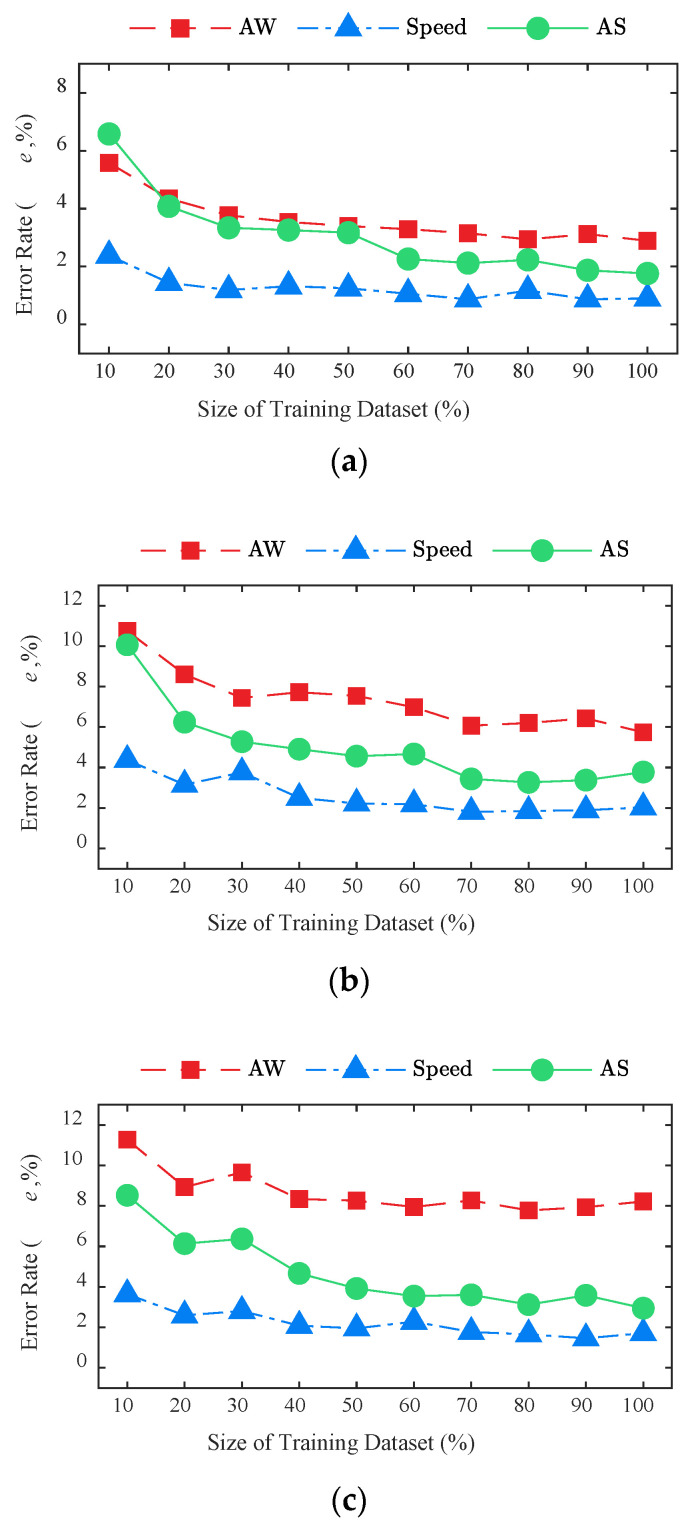
Effect of training data size on the BwimNet method’s identification performance: (**a**) 2-axle truck; (**b**) 3-axle truck; (**c**) 5-axle truck. AW, axle weight, AS, axle spacing.

Figure 16 shows the identification errors of the BwimNet method under different data sizes for 2-axle, 3-axle, and 5-axle trucks, respectively. As can be seen from Figure 16, the identification accuracy of the BwimNet method increases with the increase of the training set size and gradually reaches its saturation, indicating that increasing data size can effectively improve the performance of the BwimNet method and reduce the identification error. Specifically, due to the task of speed identifying being relatively simple, the size of the training set has a relatively small impact on speed identification, and a very low identification error of speed can be achieved when the size of the training set is 40% (1600 samples) of the original one. As for the identification of axle spacings, axle weights, and axle count, at least 70% (2800 samples) of the original training set size is needed to achieve the best identification performance.

### 4.6. Effect of Lateral Position

The influence of the lateral position has also been investigated in this paper. Truck models were set to pass the bridge at a random lateral position ranging from the left edge of lane 1 to the right edge of lane 2. Four thousand samples of strain signals of the bridge under vehicles passing along different lateral positions were recorded. The convolutional neural networks were then trained by those strain signals. Another 1000 samples were recorded and used as the test dataset to evaluate the effect of lateral loading position on the performance of the proposed method.

Table 4 illustrates the identification error of the 1000 test dataset by using the proposed method and the conventional method. Compared with the identification error in Table 3, it can be seen that the lateral position does not have much influence on the performance of the proposed method. It should be noted that lateral loading position was taken into consideration both in the training and the testing.

## 5. Discussion

In this study, a novel traffic monitoring technique named BwimNet is proposed to monitor overloading and to gather WIM statistical data for further usage. For the BwimNet, tasks of identifying vehicle information are divided into two parts and were accomplished by two well-designed neural networks: the first one (CNN-1) predicts axle counts and the second one (CNN-2) predicts speeds, axle weights, and axle spacings. One of the good points of this method is that the CNN-2 predicts traffic loads without the need for training data of vehicle weight, thus avoiding the laborious and costly data acquisition of vehicle weight. The required speed and axle spacing data can be acquired via cameras or axle detecting devices (permanent or portable), making this method convenient to be applied and promoted at a moderate cost.

Another feature of BwimNet is that, after the training process, the properties of vehicles on the target bridge can be identified by merely using the dynamic strain responses and associated strain sensors. Apparently, being free of using additional axle-detecting sensors reduces the cost of installation and maintenance of the BWIM system and avoids underlying challenges in sensor synchronization, rendering this method a potential competitive candidate for traffic load monitoring. Based on the results of parametric studies, the following findings were obtained:

(1) The BwimNet is found to be able to identify moving vehicles’ properties normally with polluted training data. Compared with the conventional method, the proposed method is much less sensitive to the errors in training data.
(2) The weights of closely-spaced axles can also be predicted with acceptable accuracy, which can hardly be identified by conventional BWIMs under the considered cases. However, methods usually tend to perform better in numerical simulations than in field tests. Further study should be conducted to evaluate the proposed method in field application.
(3) Accuracy of axle weight, axle spacing, and axle count rises with the increase of the dataset size at first and then tends to level off. The result shows that 4000 samples and 2800 samples might be sufficient for the training set of CNN-1 and CNN-2, respectively.
(4) Although the identification error of the BwimNet method may slightly increase with the deterioration of road surface condition, this method still achieved acceptable identification accuracy.

## 6. Summary and Conclusions

In this paper, the technology of an encoder-decoder structure is introduced into the traffic monitoring field. To identify the properties of moving vehicles on the target bridge, a modified encoder-decoder architecture was proposed. The new method improves the deficiencies of previous BWIM methods such as requiring additional axle detecting sensors and their relevant costly data acquisition systems and has good potential to serve as a durable and widely applicable traffic load monitoring technique.

Another advantage of this new method is that it outperforms the conventional BWIM algorithms in both stability and reliability. Comprehensive simulations and parameter studies were performed to evaluate the performance of the proposed method. Results show that good identification accuracy of vehicle properties was achieved by the proposed methodology—even under those circumstances where considerable original error exists in the training data.

Furthermore, the proposed method establishes a traffic loading monitoring framework combining the inverse problem with an unsupervised algorithm to identify the properties of moving vehicles. The encoder-decoder architecture is grafted with a signal-reconstruction layer which makes the generated embedding explainable and realizes unsupervised learning of vehicle weight prediction. Additionally, the associated modification of the encoder-decoder method is also useful for finding the solution to other inverse problems. For instance, the signal-reconstruction layer utilized in this paper can be replaced by neural networks with clear physical significance, making the embedding vector generated by the encoder explainable and finding the solution to those inverse problems.

It should be noted that site specific training data is required for each bridge when using the proposed method, because the generalization ability of neural networks trained by data collected from one particular bridge is not strong enough to cover all the other bridges with good accuracy. Fortunately, the transfer learning strategy, which is a popular trick in deep learning which uses knowledge gained from one problem to help solve another different but someway related problem (Pan and Yang 2009), can be adopted to reduce the cost of network training. Moreover, further investigation should be undertaken to investigate the performance of the proposed method on real scenario application.

## Figures and Tables

**Table 1:** Input and output data of the BwimNet method.

Input:
$\varepsilon^{m}$, time-history of measured strain response
*I(x)*, influence line of bridge response
**Output:**
*N*, axle count of vehicle *v*, velocity of vehicle **d**, vector of axle spacing (AS) $\varepsilon^{r}$, rebuilt strain response
**By-products:**
**F**, vector of axle weight (AW) GVW, gross vehicle weight

**Table 2:** Configuration of calibration truck models.

Truck Model	Configuration								
	Axle Weight (kN)					Axle Spacing (m)			
	Axle1	Axle2	Axle3	Axle4	Axle5	*l_a_*_1_	*l_a_*_2_	*l_b_*_1_	*l_b_*_2_
Two-axle truck	43.60	29.90	-	-	-	7.90			
Three-axle truck	35.60	142.10	142.40	-	-	4.27	4.27	-	-
Five-axle truck	56.70	117.00	76.40	72.90	69.40	3.00	5.10	1.10	1.10

**Table 3:** Identification errors of axle weights estimation under good RSC.

Truck	Method	Relative Error (%)					
		AW1	AW2	AW3	AW4	AW5	GVW
Two-axle truck	Moses’ algorithm	1.72	2.17	-	-	-	0.40
	BwimNet	2.27	3.51	-	-	-	0.61
Three-axle truck	Moses’ algorithm	4.16	3.26	1.96	-	-	0.32
	BwimNet	6.64	6.29	4.56	-	-	1.48
Five-axle truck	Moses’ algorithm	4.70	7.46	48.03	79.04	40.25	0.27
	BwimNet	7.55	5.76	10.62	11.86	5.76	1.30

AW = the axle weight of vehicles. GVW = the gross weight of vehicles.

**Table 4:** Identification error considering lateral loading position.

Truck	Relative Error (%)					
	AW1	AW2	AW3	AW4	AW5	GVW
Two-axle truck	2.28	3.00	-	-	-	0.65
Three-axle truck	5.11	4.61	3.99	-	-	1.03
Five-axle truck	4.97	5.66	8.89	6.90	6.18	1.26

AW, the axle weight of vehicles. GVW, the gross weight of vehicles.

## Appendix B

The noise level in percentage of white noise is defined as the ratio of root mean square value (RMS) of the noise to the RMS of the pure signal:

(A1) $$\;\sigma =\frac{\mathrm{RMS}(S_{\mathrm{noise}})}{\mathrm{RMS}(S)}.$$

The polluted signal can be obtained by the following formula:

(A2) $$\;S_{\mathrm{polluted}}=S+S_{\mathrm{noise}}=S+\mathrm{RMS}(S)\cdot N_{\mathrm{unit}}\cdot \sigma ,$$

where RMS(S) is the root mean square value of original signal, and $N_{\mathrm{unit}}$ is random samples generated from standard gauss stochastic process with zero mean and unit deviation.

## Appendix C

**Table A1:** Suggested implementation procedure of BwimNet in real application.

Stage	Operation
1. Data acquisition	a. Install one strain sensor under the soffit of each girder or slab of the target bridge for the purpose of obtaining bridge strain responses. b. Install (temporary) axle detectors (pressure sensitive sensors placed on top of the bridge, FAD sensors, or surveillance cameras) on the bridge. c. Collect training data with vehicle speed and axle spacings and relative bridge response. Then, uninstall the axle detectors if needed. d. Calculate the influence line from measured bridge response using the matrix method.
2. Network Training	e. Construct and train the CNN-1 network with bridge responses as input and axle counts as output (CNN-1 identifies the axle count of passing vehicles). f. Construct and train the CNN-2 network with axle count and true bridge responses as input, axle spacings, speed, and reconstructed bridge response as output. It should be noted that axle weights as output are the middle products of the signal reconstruction procedure in CNN-2. So, it does not need to provide weight information for the training process.
3. Predicting	g. Acquire bridge responses under the travelling vehicle to be weighed by using strain sensors. Predict axle count of the vehicle by using CNN-1. Then, feed the strain response and axle count to CNN-2 and get estimations of speed, axle spacings, and axle weights of the vehicle.
